# Intestinal Parasites and the Occurrence of Zoonotic *Giardia duodenalis* Genotype in Captive Gibbons at Krabokkoo Wildlife Breeding Center, Thailand

**DOI:** 10.3389/fvets.2019.00110

**Published:** 2019-04-17

**Authors:** Sahatchai Tangtrongsup, Duanghatai Sripakdee, Suchinda Malaivijitnond, Rungroj Angkuratipakorn, Michael Lappin

**Affiliations:** ^1^Department of Companion Animal and Wildlife Clinic, Faculty of Veterinary Medicine, Chiang Mai University, Chiang Mai, Thailand; ^2^Research Center of Producing and Development of Products and Innovations for Animal Health and Production, Chiang Mai University, Chiang Mai, Thailand; ^3^Veterinary Diagnostic Laboratory, Faculty of Veterinary Medicine, Chiang Mai University, Chiang Mai, Thailand; ^4^Department of Biology, Faculty of Science, Chulalongkorn University, Bangkok, Thailand; ^5^National Primate Research Center of Thailand, Chulalongkorn University, Bangkok, Thailand; ^6^Krabookkoo Wildlife Breeding Center, Chachoengsao, Thailand; ^7^Department of Clinical Sciences, Colorado State University, Fort Collins, CO, United States

**Keywords:** intestinal parasites, *Giardia duodenalis*, captive, gibbons, Thailand

## Abstract

Intestinal parasitic infections can have an impact on health and growth of wildlife. The current study aims were to determine the prevalence of intestinal parasites and to molecular characterize *Giardia duodenalis* and *Cryptosporidium* spp. in captive gibbons at Krabokkoo Wildlife Breeding Center, Thailand. Fifty-five gibbons, 2 agile- (*Hylobates agilis*), 38 lar- (*Hylobates lar*) and 15 pileated gibbons (*Hylobates pileatus*) were included in this study. Fecal samples were collected individually at Krabokkoo Wildlife Breeding Center, Chachoengsao province, eastern Thailand, in November 2013. Intestinal parasitic infections were examined by zinc sulfate centrifugation flotation and by a commercially available immunofluorescent assay (IFA) for detection of *G. duodenalis* and *Cryptosporidium* spp.. Polymerase chain reaction targeting the *Giardia* glutamate dehydrogenase (gdh), beta- giardin (bg), triose phosphate isomerase (tpi) genes, and the *Cryptosporidium* small subunit-rRNA and heat-shock protein (hsp70) following by DNA sequencing were performed on the IFA positive samples. The overall prevalence of intestinal parasitic infection in gibbons at Krabokkoo Wildlife Breeding Center was 12.7% (95%CI: 5.3–24.5), *Strongyloides* spp. eggs or larvae were present in all positive samples. Co-infections with *G. duodenalis* were detected in 1.8% (95%CI: 0.1–9.7) of the samples. Based on the sequencing results of the three genes, the IFA *Giardia* positive isolate typed as the zoonotic genotype B. Since the data reveals the occurrence of zoonotic *Giardia* genotype, good hygiene management is suggested to prevent the transmission of this pathogen from gibbon to human, and vice versa.

## Introduction

Intestinal parasitic infections are the most common causes of gastrointestinal diseases in captive wildlife. These infections can cause a wide range of clinical signs, from subclinical infections to malabsorption, abdominal pain, diarrhea, vomiting, anemia, severe dehydration, and death (1–3). As the living area is limited, stress and other factors such as artificial environment, poor diet or the presence of humans lead to the high risk of infection and weaken the natural resistance of the host, making the clinical illness possible (4). The weakened health condition of these captive animals can have a negative impact on their reproduction which is of major concern in the zoos and wildlife breeding facilities of captive or endangered species (3, 5).

Several studies on helminthic parasites in the free-ranging (5–10) and captive populations (4, 11–15) of non-human primates (NHP) have been conducted worldwide and they reported a high prevalence of intestinal parasites. For example, the prevalence of endoparasites in western lowland gorillas at Bai Hokou, Dzangha-Ndoki National Park, Central African Republic has been reported to be up to 100% (7). Of all intestinal parasites detected in NHP, *Strongyloides* spp.*, Oesophagostomum* spp., *Trichuris* spp., *Ascaris* spp., and hookworms were the most common intestinal parasites.

Eight assemblages (A-H) of *Giardia duodenalis* and at least 27 *Cryptosporidium* spp. have been described (16, 17). Infection with *G. duodenalis* and *Cryptosporidium* ssp. in NHP are common (18–21). In wild and captive NHP, prevalence rates of these infections range from undetectable level to as high as 70% (20, 22–26). In several studies on NHP, zoonotic assemblages of *G. duodenalis*, assemblage A and B, were identified and the assemblage B was more prevalent in both captive and free-range animals (18, 23, 27, 28). *Cryptosporidium hominis* and *C. parvum* were commonly identified in *Cryptosporidium-*infected primates (29–31).

Currently, there is no information available regarding intestinal parasitic infection in captive gibbons in breeding facilities in Thailand. Knowing background prevalence of gastrointestinal parasites can be beneficial in the health management program in gibbons for the reproduction at the Krabokkoo Wildlife Breeding Center. The aims of this study were, therefore, to determine the prevalence of intestinal parasites and to molecular characterize *Giardia duodenalis* and *Cryptosporidium* spp. isolates to determine the potential of zoonotic transmissions of these pathogens from captive gibbons at Krabokkoo Wildlife Breeding Center, Thailand.

## Materials and Methods

### Study Area

**Figure 1:**
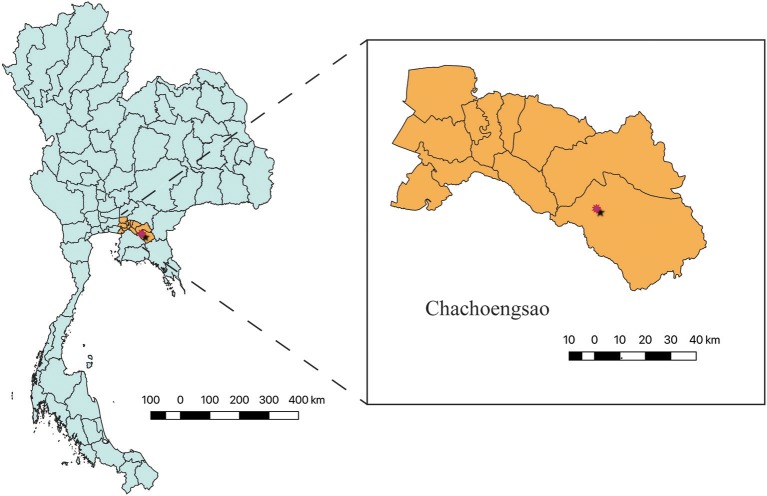
Krabokkoo Wildlife Breeding Center is located in Chachoengsao province, eastern Thailand at 13°28′5.05″N, 101°35′37.30″E, 2.5 h from Bangkok.

Krabokkoo Wildlife Breeding Center is located in Chachoengsao province, eastern Thailand, at the coordinates of 13°28′5.05″N, 101°35′37.30″E (Figure 1) and at 47 meters above the sea level. In November 2013, this facility accommodated four species of gibbons, 65 white-handed (*Hylobates lar*), 15 pileated (*Hylobates pileatus*), 2 agile (*Hylobates agilis*), and 2 crown (*Nomascus* spp.) gibbons. The gibbons were separated among species and were housed individually or in groups. Siblings or a family were housed together. They were fed with vegetable- and fruit-based diet and water was supplied in a bowl. Drinking water was replaced on a daily basis. All gibbons were dewormed every 3 months. The temperature are 23–27°C in winter (mid-October–mid-February), 35–40°C in summer (mid-February–mid-May), and 28–35°C in rainy season (mid-May–mid-October) (32).

### Fecal Sample and Data Collection

Fifty-five fecal samples were collected from white-handed (*H. lar*) (*n* = 38), pileated (*H. pileatus*) (*n* = 15), and agile (*H. agilis*) (*n* = 2) gibbons during the November of 2013. The samples used in the study were part of the study on genetic diversity of macaques and Hylobatidae gibbons in Thailand. Each fecal sample was collected from the ground (care was taken not to have soil contamination), kept in a labeled plastic bag and stored at 4°C until examination. Sex, age, cage and identification number were recorded at the time of collection. Fecal samples were shipped on ice to the Faculty of Veterinary Medicine, Chiang Mai University, Chiang Mai, Thailand within a week and the fecal consistencies were determined upon arrival.

## Diagnostic Procedures

### Microscopic Examination of Fecal Samples After Zinc Sulfate Centrifugal Flotation and IFA for *Giardia duodenalis* and *Cryptosporidium* spp. Detection

Fecal samples were examined for the presence of intestinal parasitic eggs, larvae, protozoal cysts and oocysts using microscopic examination after zinc sulfate centrifugal flotation (33). *Giardia duodenalis* and *Cryptosporidium* spp. infections were determined using a commercially available direct immunofluorescent assay (IFA) (MeriFluor® *Cryptosporidium/Giardia* Test Kit, Meridian Diagnostic Corporation, Cincinnati, OH). Prior IFA, the fecal samples (3 grams) were concentrated using sucrose gradient centrifugation technique as previously described (34). IFA was carried out according to the manufacturer's instruction.

### DNA Isolation and Molecular Detection of *Giardia duodenalis* Infection

Three hundred microliters of each *Giardia* or *Cryptosporidium* IFA positive fecal concentrate were subjected to DNA extraction using the FastDNA® kit (MP Biomedicals, Solon, OH, USA) following an established protocol (35).

PCR assays targeting *Giardia* glutamate dehydrogenase (gdh), beta-giardin (bg), and triosephosphate isomerase (tpi) genes, and *Cryptosporidium* heat-shocked protein (hsp70), and small subunit ribosomal RNA (SSU-rRNA) were used for molecular characterization of the respective organisms in the IFA positive samples. Previously described PCR protocols were used (36–42).

### DNA Sequencing and Phylogenetic Analysis

The PCR products were purified using QIAquick Gel Extraction Kit (Qiagen, OH, USA) and purified PCR product was evaluated by nucleotide sequencing using a commercially available service (1st Base Laboratory, Selangor, Malaysia). For each target gene, the obtained sequences from both directions were aligned and a consensus sequence was generated and compared with nucleotide sequences from the nucleotide database from the GenBank using BLAST analysis (http://blast.ncbi.nlm.nih.gov/Blast.cgi). Phylogenetic and molecular analyses were conducted using the MEGA 6.06 program (43). Multiple sequence alignments were performed using MUSCLE (44), and the phylogenetic analyses were performed by the Maximum Likelihood method based on the Kimura 2-parameter model. The consensus tree was obtained after bootstrap analysis with 500 replications. Reference strains of the different assemblages were retrieved from the GenBank and included for comparative phylogenetic analyses.

### Statistical Analyses

A sample was considered positive for gastrointestinal parasites if parasitic eggs or larvae were detected by light microscopic examination after zinc sulfate centrifugal flotation. A sample was considered positive for *Giardia* and *Cryptosporidium* if at least one (oo)cyst was detected by either microscopic examination or IFA. Gibbons were grouped by species, age (<10 years, ≥10 years), and sex. Overall prevalence and 95% confidence interval (95%CI) were calculated. Associations of age category, sex, fecal consistency, gibbon species, and parasitic infestation results were analyzed using Fisher's Exact test. A *P* < 0.05 was considered statistically significant. All statistical analyses were performed using STATA statistical software release 10.1 (Stata Corp., College Station, Texas, USA).

## Results

### Microscopic Examination of Fecal Samples After Zinc Sulfate Centrifugal Flotation and IFA for *Giardia duodenalis* and *Cryptosporidium* spp. Infections

Characteristics of gibbons and samples and the descriptive statistics are shown in Table 1. Of 55 fecal samples, *Strongyloides* spp. eggs or larvae were detected in 7 samples by microscopic examination after zinc sulfate centrifugal flotation. *Giardia* cysts were detected in one fecal sample by IFA. *Cryptosporidium* oocysts were not detected by IFA in any fecal samples, therefore, PCR assays were not performed.

**Table 1:** Prevalence of intestinal parasitic infection by gibbon species, age, sex, and fecal consistency.

	*Strongyloides* spp. % (95%CI^*^)	*Giardia duodenalis* % (95%CI^*^)
Overall (55)	12.73 (5.27–24.48)	1.80 (0.05–9.71)
**SPECIES**		
*Hylobates agilis* (2)	0.00 (0.00–84.19)^†^	0.00 (0.00–84.19)^†^
*Hylobates lar* (38)	15.79 (6.02–31.25)	2.63 (0.07–13.81)
*Hylobates pileatus* (15)	6.67 (0.17–31.95)	0.00 (0.00–21.80)^†^
**AGE**		
<10 years (6)	0.00 (0.00–19.51)^†^	0.00 (0.00–45.93)^†^
≥ 10 years (17)	16.67 (0.42–64.12)	0.00 (0.00–19.51)^†^
Unknown (32)	18.75 (7.21–36.44)	3.13 (0.08–16.22)
**SEX**		
Female (28)	17.86 (6.06–36.89)	3.57 (0.09–18.34)
Male (27)	7.41 (0.91–24.29)	0.00 (0.00–12.77)^†^
**FECAL CONSISTENCY**		
Formed or soft (49)	12.24 (4.63–24.77)	2.04 (0.05–10.85)
Diarrhea (6)	16.67 (0.42–64.12)	0.00 (0.00–45.93)^†^

*A number in parentheses represents the number of samples in each category*.

* 95% Confidence Interval

† *One sided 97.5%CI*.

### *Giardia duodenalis* Sequences and Phylogenetic Analyses

**Figure 2:**
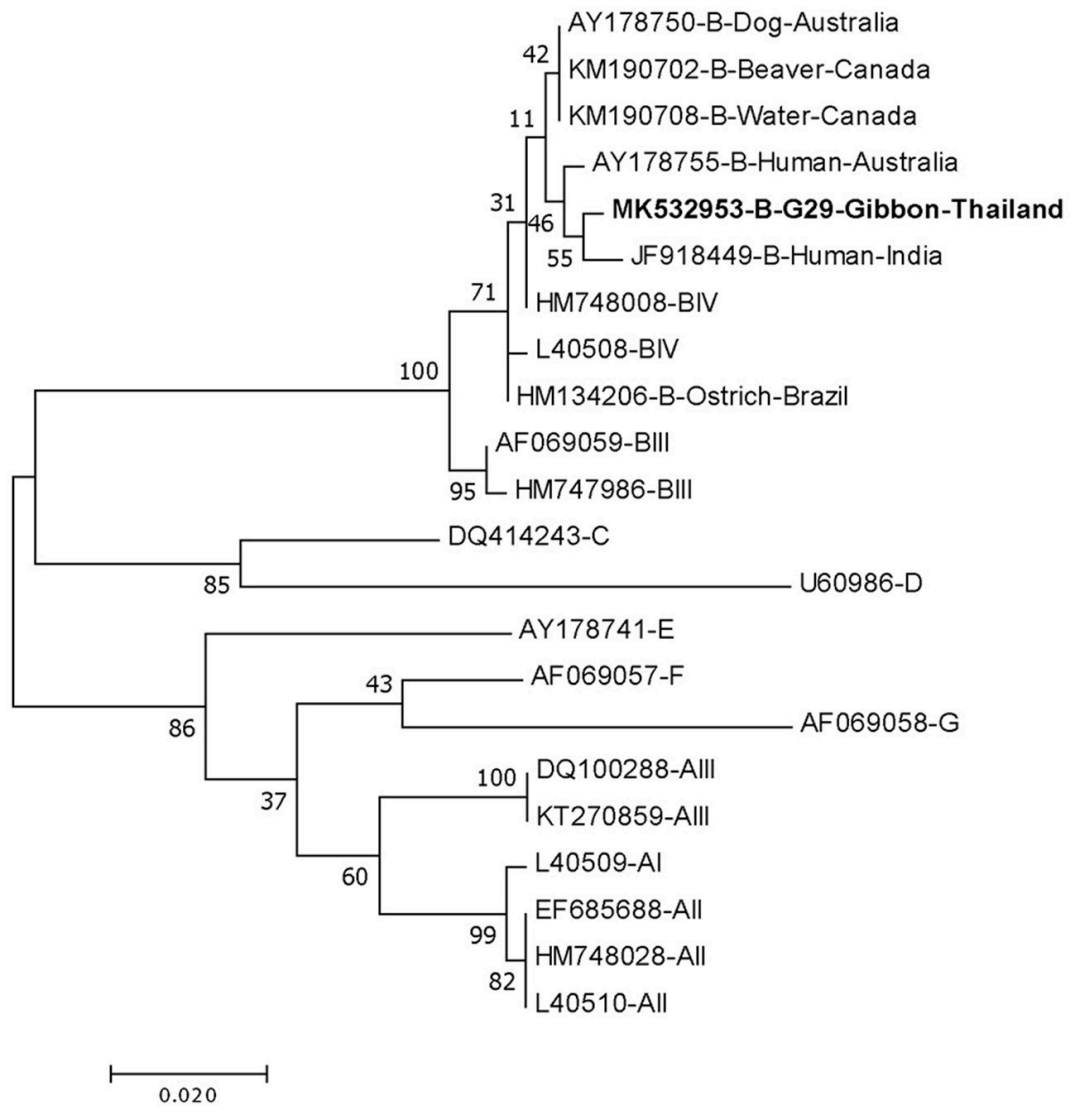
Phylogenetic tree of *Giardia* isolate based on the sequence of glutamate dehydrogenase (gdh) gene from a gibbon in this study by the Maximum Likelihood algorithm using the MEGA 6.06 program. Sequences obtained from GenBank are indicated by their accession numbers. Percentage bootstrap supports (500 replicates) are shown by numbers at the respective nodes. Bold texts represent the *Giardia* detected in this study.

**Figure 3:**
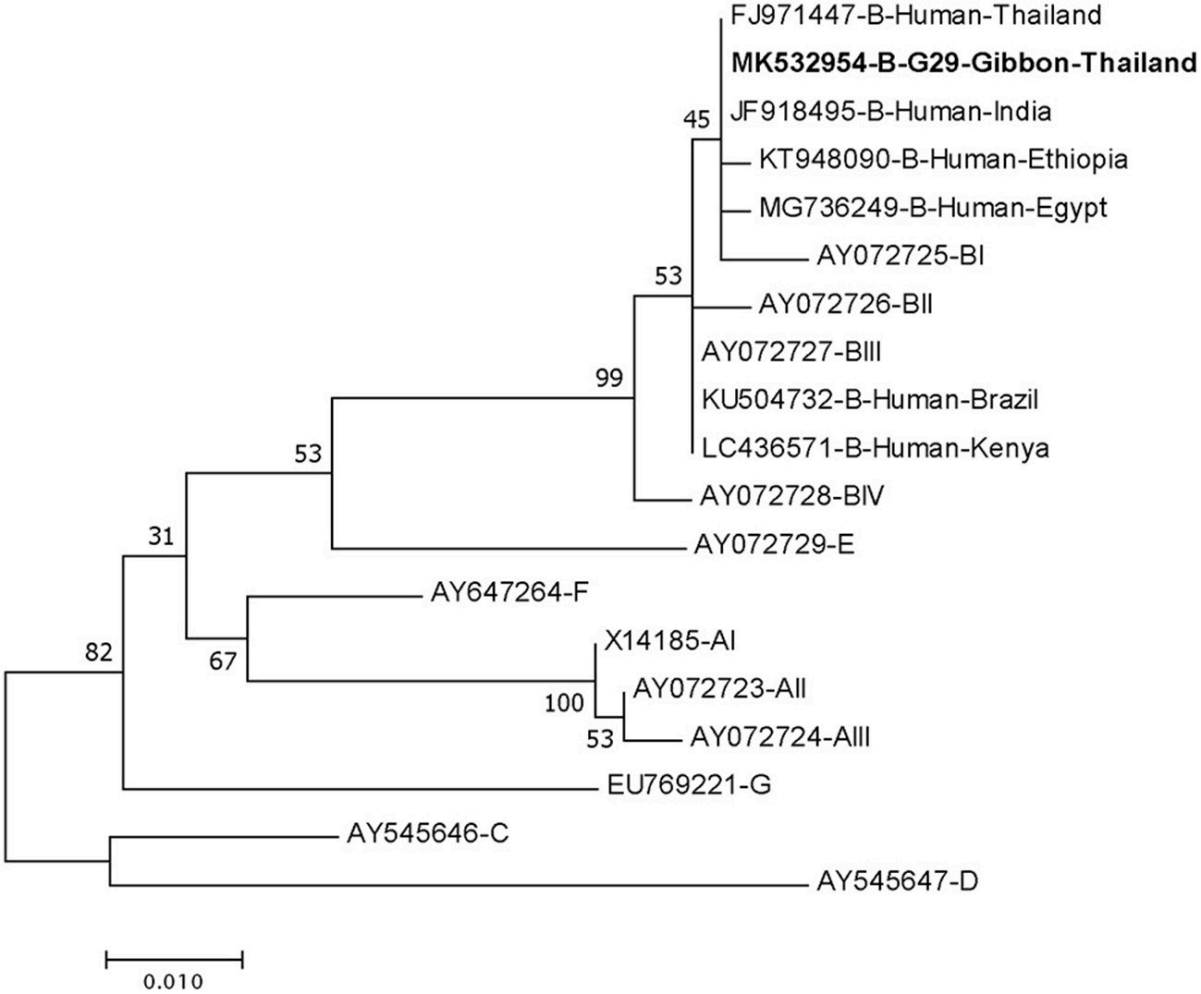
Phylogenetic tree of *Giardia* isolate based on the sequence of beta-giardin (bg) gene from a gibbon in this study by the Maximum Likelihood algorithm using the MEGA 6.06 program. Sequences obtained from GenBank are indicated by their accession numbers. Percentage bootstrap supports (500 replicates) are shown by numbers at the respective nodes. Bold texts represent the *Giardia* detected in this study.

**Figure 4:**
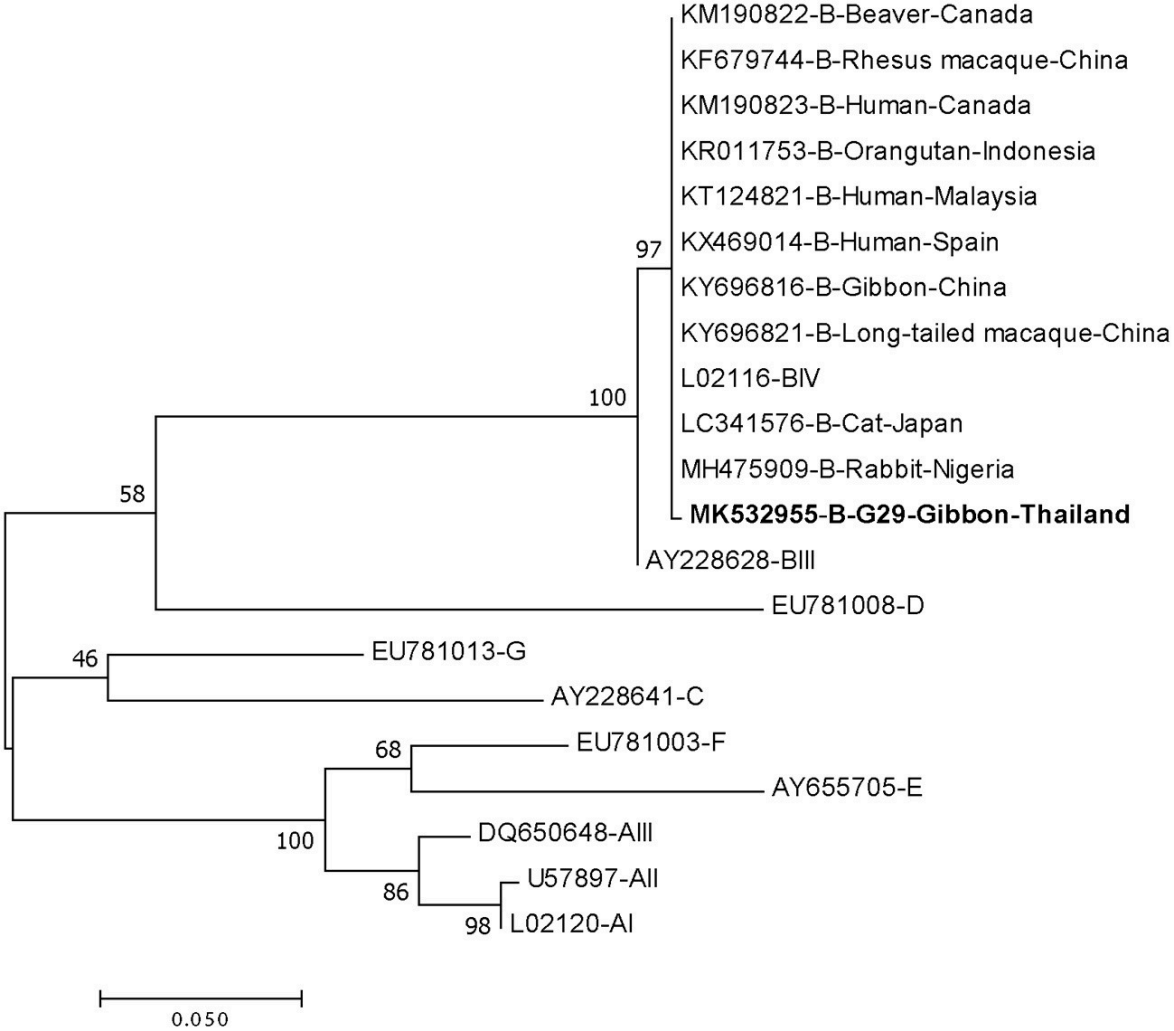
Phylogenetic tree of *Giardia* isolate based on the sequence of triose phosphate isomerase (tpi) gene from a gibbon in this study by the Maximum Likelihood algorithm using the MEGA 6.06 program. Sequences obtained from GenBank are indicated by their accession numbers. Percentage bootstrap supports (500 replicates) are shown by numbers at the respective nodes. Bold texts represent the *Giardia* detected in this study.

DNA fragments of the only IFA *Giardia* positive sample (G29) were successfully amplified and typed as assemblage B by the three genes (gdh, bg, and tpi). The gdh sequence of G29 showed 99% homology to the assemblage B gdh sequences recovered from a water sample and a beaver in Canada, an ostrich in Brazil, a human and a dog in Australia, and a human from India (Figure 2). The G29 gdh sequence has 3 SNPs (single-nucleotide polymorphism) at position 12 (A vs. T), 93 (A vs. G), and 199 (A vs. G), when compared to those sequences mentioned previously; however, neither of these SNPs resulted in amino acid change. The beta-giardin sequence of G29 showed 100% homology to the assemblage B from human from Thailand and India and 99.9% homology to assemblage B human isolates from Kenya, Egypt, Brazil, and Ethiopia (Figure 3). Sequences from tpi gene contained ambiguous nucleotides at position 108 (T or G) and 443 (A or T). When translating the G29 tpi sequence to amino acids, substitution of T with G at position 108 did not cause amino acid change, whilst substitution of A with T at position 443 resulted in an amino acid change from Valine to Aspartic acid. Variants of tpi sequences showed 99–100% homology to the tpi sequences recovered from rhesus macaque, long-tailed macaque, and gibbons from China, Sumatran Orangutan from Indonesia, beaver from Canada, cat from Japan, rabbit from Nigeria, and humans from Canada, Malaysia, and Spain (Figure 4). From the phylogenetic analyses of gdh, bg and tpi genes, the *G. duodenalis* isolate in this study was placed into BIV, BI, and BIV branch, respectively (Figures 2–4).

### Statistical Analyses

Due to the low detection of the parasites and the small sample size, the power to detect associations between any risk factors and infections was insufficient.

## Discussion

The current study represents the first report of the intestinal parasites as well as *G. duodenalis* and *Cryptosporidium* spp. prevalence rates and *Giardia* genotypes in captive gibbons in Thailand. The prevalence of these infections were commonly high and ranged from 25 to 100% in either free-ranging (5, 7–10, 22, 28, 31, 45, 46) or captive non-human primates (4, 7, 11, 13–15, 20, 23–26, 46, 47). In the current study, overall prevalence rates of nematodes, *G. duodenalis*, and *Cryptosporidium* spp. in gibbons were 12.7, 1.8, and 0%, respectively. These prevalence rates, however, were comparable to the previous report of 0–16.4% in gibbons in zoological parks in China (48). The low prevalence in this study may be from collecting a single fecal sample from each animal. Parasitic eggs, *Giardia* cysts and *Cryptosporidium* oocysts are intermittent shed in the feces, therefore, three or more fecal samples from animals can increase the sensitivity of intestinal parasites (33). In addition, the low detection rate of *Giardia* and the lack of *Cryptosporidium* spp. were also because of the number of cysts/oocysts were below the detection limit of the diagnostic tests used in the study (49).

The most common helminthic species detected in NHP were *Strongyloides* spp., *Trichuris* spp., *Oesophagostomum* spp., *Ascaris* spp. and hookworms (5, 7, 22, 45, 46). A similar pattern of gastrointestinal parasites was also observed in captive gibbons in a zoo in China (13). In a study in 23 wild white-handed gibbons at Khao Yai National Park, Thailand, *Trichuris* spp. and *Ternidens* spp. were the most prevalent helminthic parasites detected (91.3%), followed by *Strongyloides fuelleborni* (56.5%) (9). However, in the current study, only *Strongyloides* spp. eggs or larvae were detected in the fecal samples. *Strongyloides* spp. are soil-transmitted nematodes with an estimated 370 million people infected worldwide (50). In this study, the detection of *Strongyloides* spp. in feces is less likely to be from contaminated soil as fecal samples were carefully collected not be contaminated with soil before the storage in a plastic bag. These nematodes can cause a chronic and persistent strongyloidiasis in the infected host because of the autoinfective life cycle (51) and cause diarrhea, hyperinfection syndrome, dissemination, and death in immunocompromised hosts. *Strongyloides stercoralis* is a primary species infecting human; however, the infections of primates' parasites *S. fuelleborni fuelleborni and S. fuelleborni kellyi* have also been reported (8). The molecular characterization of *Strongyloides* positive samples is suggested since microscopic identification is insufficient for species identification and determination of its zoonotic potential. In this study, the species identification was not performed but this finding has raised concerns regarding the zoonotic potential. Since the fatal strongyloidiasis cases of gibbons in Thailand has been reported (1) and *Strongyloides* spp. is also an important parasitic helminth of humans (8), an effective anthelminthic program is recommended.

*Giardia duodenalis* and *Cryptosporidium* spp. are important intestinal protozoans in non-human primates. These pathogens can cause a wide range of clinical signs, from subclinical to malabsorption, abdominal pain, failure to thrive, acute or chronic diarrhea especially in young, old and immune-compromised animals (3, 52, 53). The organisms are commonly found in both free-ranging and captive non-human primates with the prevalence from 0–70% to 0–48%, for *Giardia* and *Cryptosporidium* infections, respectively (19, 21, 22, 24, 31, 47, 54). In Thailand, a low prevalence rate (1/23, 4.35%) of *Cryptosporidium* spp. infection has been previously reported in wild white-handed gibbons at Khao Yai National Park in Thailand. IFA was used for the detection of *Giardia* cysts or *Cryptosporidium* oocysts in repeatedly collected fecal samples that ranged from 3 to 25 samples per gibbon, resulting in a total of 324 samples (9). In that study, there was no *Giardia* detected. In this study, in contrast, no *Cryptosporidium* oocysts were detected in all fecal samples and *Giardia* cysts were detected in only one fecal sample of 55 samples. These findings could be due to that the numbers of cysts or oocysts of these pathogens were low and were below the detection limit of the IFA tests. The *Giardia* positive sample, in this study, typed as assemblage BIV and BI by gdh and tpi and bg genes, respectively. This finding is in agreement with previous reports that assemblage B was predominant in NHP (18, 23, 27, 28). Although the prevalence of *Giardia* infection in this study is low, the identification of *G. duodenalis* assemblage B may suggest the potential of zoonotic or anthroponotic transmissions in this area.

The limitations of this study are the small sample size and the nature of single sample collected from each animal; selection bias may have led to an underestimation of the prevalence rates. A larger sample size or more frequent collection of gibbon's feces are needed for further studies. This study had inadequate power to detect associations between any risk factors and infections. In addition, we analyzed gibbons' species, age, sex, and diarrhea status; however, other important risk factors, e.g., season, diet, or water source could be suggested for future study to help in prevention and control of intestinal parasitic infection in this population.

## Author Contributions

ST, SM, and ML designed the study. SM and RA performed fecal sample collection. DS performed the microscopic fecal examination, ST performed IFA and molecular analyses. ST analyzed sequences. ML provided laboratory supplies. ST interpreted the results and wrote the manuscript. All authors read and approved the final manuscript.

### Conflict of Interest Statement

The authors declare that the research was conducted in the absence of any commercial or financial relationships that could be construed as a potential conflict of interest. The reviewer AS declared a shared affiliation, with no collaboration, with one of the authors, ML, to the handling editor at time of review.
